# Selective serotonin reuptake inhibitor use and breast cancer survival: a population-based cohort study

**DOI:** 10.1186/s13058-017-0928-0

**Published:** 2018-01-19

**Authors:** John Busby, Ken Mills, Shu-Dong Zhang, Fabio Giuseppe Liberante, Chris R. Cardwell

**Affiliations:** 1 Centre for Public Health, Queen’s University Belfast, Belfast, UK; 2 Centre for Cancer Research and Cell Biology (CCRCB), Queen’s University Belfast, Belfast, UK; 3 Northern Ireland Centre for Stratified Medicine, Biomedical Sciences Research Institute, University of Ulster, C-TRIC Building, Altnagelvin Area Hospital, Londonderry, UK

**Keywords:** Selective serotonin reuptake inhibitors, Breast cancer, Progression, Survival, Pharmacoepidemiology

## Abstract

**Background:**

Nearly 50% of breast cancer patients suffer from depression or anxiety. Selective serotonin reuptake inhibitors (SSRIs), the first-line pharmacological treatment for depression, have been implicated in breast cancer development through increased prolactin levels and tamoxifen metabolism inhibition. Previous studies of breast cancer progression have focused on tamoxifen users, or have been limited by their small sample size and methodology. Therefore, we used UK population-based data to more robustly investigate the association between SSRI use and cancer-specific mortality.

**Methods:**

A cohort of patients with newly-diagnosed breast cancer between 1998 and 2012 was selected from English cancer registries and linked to prescription records from the Clinical Practice Research Datalink, and to death records from the Office for National Statistics. We used Cox regression models to calculate hazard ratios (HRs) comparing mortality between post-diagnostic SSRI users and non-users (using time-dependant covariates), after adjusting for demographics, comorbidities and pre-diagnosis use of hormone replacement therapy or oral contraceptives. We conducted several additional analyses to assess causality.

**Results:**

Our cohort included 23,669 breast cancer patients, of which 2672 used SSRIs and 3053 died due to their breast cancer during follow-up. After adjustment, SSRI users had higher breast cancer-specific mortality than non-users (HR = 1.27; 95% confidence interval (CI) 1.16, 1.40). However, this association was attenuated when restricting to patients with a prior history of depression (HR = 1.14; 95% CI 0.98, 1.33), and when comparing to users of other antidepressant medications (HR = 1.06; 95% CI 0.93, 1.20). There was some evidence of higher mortality among long-term SSRI users, even when restricting to patients with prior depression (HR = 1.54; 95% CI 1.03, 2.29).

**Conclusions:**

In this large breast cancer cohort, SSRI use was associated with a 27% increase in breast cancer mortality. The cause of this is unknown; however, confounding by indication seems likely as it was largely attenuated when restricting to patients with prior depression, or when comparing SSRIs to other antidepressant medications. Clinicians should not be unduly concerned when prescribing SSRIs to breast cancer patients, but the increase in mortality among long-term SSRI users warrants further investigation.

**Electronic supplementary material:**

The online version of this article (doi:10.1186/s13058-017-0928-0) contains supplementary material, which is available to authorized users.

## Background

Breast cancer is the second most common cancer in the world, with 1.7 million new cases diagnosed annually [1, 2]. In England, around 15% of patients die due to the disease within 5 years [3]. Patients also suffer markedly reduced quality of life, and substantially higher healthcare costs during treatment and recovery [4–7]. Breast cancer patients are highly susceptible to mental health problems with nearly 50% suffering depression or anxiety [8], and 10–20% experiencing a major depressive episode after diagnosis [9, 10].

Selective serotonin reuptake inhibitors (SSRIs) are a group of antidepressant medications recommended for moderate/severe depression and treatment-resistant mild depression [11]. In England, 36 million prescriptions were dispensed during 2016, representing a 6% rise on the previous year [12]. Increasing SSRI prescriptions have also been observed in several other countries including the USA, Denmark and Spain [13, 14]. SSRIs are widely used among breast cancer patients, mainly to treat depression, but also to control hot flushes [15, 16].

Despite their widespread use, there have long been concerns that SSRIs may promote breast cancer by increasing prolactin levels [17–19], an accepted risk factor for tumour progression [20–22]. More recently, SSRIs have been shown to increase the rate of brain metastases in breast cancer mouse models, by altering the permeability of the blood–brain barrier [23]. SSRIs may also affect cancer outcomes by interfering with tamoxifen metabolism through inhibition of the CYP2D6 enzyme [24, 25]. In humans, several studies have investigated the association between SSRIs and breast cancer risk, although these have reached inconsistent conclusions [26–28]. Studies of breast cancer progression are rarer, and have generally focused on patients treated with tamoxifen [29, 30]. Only three studies have considered a broader population of breast cancer patients, and these were limited by their small size and/or other methodological weaknesses (e.g. self-reported medication use) [31–33]. Consequently, we used routine data from the UK to more robustly assess the association between SSRI use and mortality among a population-based cohort of breast cancer patients.

## Methods

### Data sources

Our study used data from the English National Cancer Data Repository (NCDR), linked to GP records from the UK Clinical Practice Research Datalink (CPRD), deprivation indices from census information and death registration data from the Office for National Statistics (ONS). The NCDR holds UK-wide data from English cancer registries compiled from general practices, NHS and private hospitals, and death certificates [34]. It contains detailed information about the patient’s cancer, including diagnosis year, stage, histologic grade, tumour histology and treatment (surgery, chemotherapy and radiotherapy). The CPRD contains computerised medical records from 674 general practices (approximately 7% of the UK population) which are audited for data completeness and quality [35]. Practices meeting a predefined quality threshold are deemed ‘up to research standard’ and included in future extracts. Data recorded within the CPRD include patient demographics, clinical diagnoses (using Read codes) and prescription medication use. Previous research has found CPRD prescription and clinical information to be of high quality [35–37]. ONS death-registration data provide details on the date and cause(s) of death.

### Study design and population

We used the NCDR to identify a cohort of patients with newly-diagnosed breast cancer (ICD code C50) between 1998 and 2012. Cohort members with a previous record of cancer were identified and excluded from the analysis using a list of cancer Read codes modified for use in the CPRD [38]. Patients were excluded if they were diagnosed: (a) before they were registered with a CPRD practice, (b) before their practice was deemed up to research standard, (c) after they left a CPRD practice or (d) after data were last collected from their practice by the CPRD. A small number of patients were recorded within the NCDR more than once; when this occurred we used their first record. Patients with stage zero breast cancer (ductal carcinoma in situ) were also excluded.

Deaths were identified from ONS records, and breast cancer-specific deaths were defined as those with a primary cause of breast cancer (ICD code C50). Patients who died within the first year of the study were excluded as it is unlikely that these could be influenced by post-diagnostic medication use, therefore the follow-up period started from 1 year after diagnosis. The end of follow-up was the earliest date of death, end of registration with the practice, last collection of data from the practice or end of death record follow-up.

### Definition of exposure

We used the British National Formulary to compile a list of proprietary and generic medication names for SSRIs (Additional file 1: Appendix 1). We added a lag of 12 months to SSRI use as these medications are unlikely to have an immediate effect on breast cancer progression, and to prevent reverse causation [39, 40]. A diagram illustrating our design is shown in Additional file 1: Appendix 2. We defined patients as users if they had at least one prescription during the exposure period. To enable the testing of dose–response relationships we extracted data on the medication prescribed, number of packs/tablets and medication strength, and calculated a defined daily dose (DDD) for each prescription. The DDD system is a validated measure of drug consumption maintained by the World Health Organisation [41]. A single DDD is the average maintenance dose per day of a drug used for its main indication in adults (e.g. depression for SSRIs). There was insufficient information to calculate DDDs for 0.1% of prescriptions, and implausible values were recorded in a further 0.7%. In these cases we assumed the most common DDD based on other prescriptions with complete information. We calculated a running DDD total for each patient and identified the day when patients received their first (first use), 365th (1-year use), 1095th (3-year use) and 1825th (5-year use) DDD.

### Covariates

Patients’ age, smoking, alcohol and obesity (BMI > 30) data were determined from the closest GP record before breast cancer diagnosis (values more than 10 years before diagnoses were discarded). We used GP records to identify pre-diagnosis comorbidities (cerebrovascular disease, chronic pulmonary disease, congestive heart disease, diabetes, liver disease, myocardial infarction, peptic ulcer disease, peripheral vascular disease, renal disease) using a list of Read codes modified for use in the CPRD [38]. We also identified post-cancer diagnosis records of hot flushes (Read codes 1657, 1662, 1662.12, 2223, K5A2000, K5A2011, R008100, R008400) as these have been associated previously with breast cancer outcomes [42], and SSRIs are sometimes used for this indication [15].

Deprivation data were available from census information and based on the 2010 Index of Multiple Deprivation (IMD) score of the patient’s postcode. We used CPRD prescription records to identify patients who received hormone therapy treatment (tamoxifen or aromatase inhibitors) after diagnosis, and those who had used oral contraceptives or hormone replacement therapy (HRT) prior to diagnosis, as these have been shown previously to influence breast cancer development [43, 44].

### Statistical analysis

We calculated descriptive statistics and compared the demographic and clinical characteristics of the SSRI users and non-users. We used time-dependent Cox regression models to calculate hazard ratios (HRs) comparing breast cancer-specific death between SSRI users and non-users. In our primary analysis we included SSRI use as a time-varying covariate to avoid immortal time bias [45]. Therefore patients were initially included within the analysis as non-users until 12 months after their first use (due to the exposure lag), after which they were included as users. Our primary analysis adjusted for age at diagnosis, year of diagnosis, deprivation quintile, comorbidities (separate terms for each), hot flushes, prior use of HRT or oral contraceptives, and treatment within 6 months of diagnosis (separate terms for surgery, chemotherapy, radiotherapy, tamoxifen, aromatase inhibitors). We repeated our analysis by the number of DDDs prescribed (e.g. patients were included in the 1–364 DDD group until 12 months after they received their 365th DDD), and for each commonly prescribed SSRI medication (≥ 2% use in cohort).

### Sensitivity and subgroup analyses

We conducted sensitivity analysis for all-cause mortality, and for cause-specific mortality where deaths with a secondary cause of breast cancer were included. We also conducted sensitivity analysis with a lag period of 6 months (patients followed-up from 6 months after diagnosis) and 2 years (patients followed-up from 2 years after diagnosis). We performed two simplified analyses which controlled for immortal time bias without requiring time-varying covariates [45]. Firstly, we based SSRI use on the year after diagnosis. Secondly, we based SSRI use on the year prior to diagnosis, and followed-up patients from the date of diagnosis. Diagrams illustrating the design of our sensitivity analyses which vary the exposure lag and/or period are given in Additional file 1: Appendix 2. We conducted a separate analysis stratified by tamoxifen use as previous studies have indicated that SSRIs can interfere with tamoxifen metabolism [29]. Patients were split into tamoxifen non-users, users and adherent users (defined as being prescribed at least 150 DDD in the 6 months after cancer diagnosis).

To ensure that confounding by indication was not driving our results we conducted two further sensitivity analysis restricted to patients with more similar diagnoses. First, we restricted our analysis to patients with a diagnosis of depression (using Read codes from previous work [46]) or record of antidepressant prescription (SSRIs, tricyclic antidepressants, monoamine-oxidase inhibitors, agomelatine, duloxetine, flupentixol, mirtazapine, reboxetine, venlafaxine, vortioxetine), in the year prior to breast cancer diagnosis. Second, we compared patients who received SSRIs to those who received a different antidepressant medication after diagnosis (using a time-varying covariate), as the use of an active comparison can overcome several common pharmacoepidemiological biases [47]. Similarly, we conducted negative control analyses for tricyclic antidepressants and venlafaxine to identify confounding [48], as they have a much weaker effect on prolactin levels [49] and do not interact with tamoxifen metabolism [50, 51] but have similar indications to SSRIs.

We performed additional sensitivity analysis adjusting for tumour prognostic features (stage, grade) and patient lifestyle factors (smoking, alcohol consumption, obesity) using complete-case and multiple imputation with chained equations (MICE). The MICE imputation used ordered logit models with age, deprivation, death indicator and the baseline hazard function as covariates [52]. Briefly, MICE is a simulation-based approach for handling missing data which leads to valid statistical inferences under certain circumstances [53]. Lastly we repeated our analysis but omitted ‘previous hot flush diagnosis’ from the model.

## Results

### Cohort description

We identified 27,008 breast cancer cases with no prior cancer diagnosis registered at CPRD practices. We excluded 3339 patients from the analysis as they had less than 12 months follow-up (*n* = 3189), stage zero cancer (*n* = 135) or a duplicate record in the NCDR (*n* = 15), leaving 23,669 patients for analysis. Median follow-up was 5.5 years (maximum 17.8 years). SSRI users were more likely to be younger, from a deprived area, have comorbidities, have previously received hormone replacement therapy or oral contraceptives, be treated with chemotherapy and be current smokers (Table 1).

**Table 1:** Patient characteristics by SSRI use

	All patients	SSRI use	
		Non-user	User
Number of patients	23,669	18,885	4784
Year of diagnosis			
1998–2002	5738 (24.2%)	4491 (23.8%)	1247 (26.1%)
2003–2007	8776 (37.1%)	6911 (36.6%)	1865 (39.0%)
2008–2012	9155 (38.7%)	7483 (39.6%)	1672 (34.9%)
Mean age at diagnosis (SD)	61.9 (14.0)	62.6 (13.9)	58.9 (13.9)
0–49	4774 (20.2%)	3479 (18.4%)	1295 (27.1%)
50–59	5913 (25.0%)	4590 (24.3%)	1323 (27.7%)
60–69	5955 (25.2%)	4903 (26.0%)	1052 (22.0%)
70–79	4037 (17.1%)	3357 (17.8%)	680 (14.2%)
Deprivation quintile			
1 (least deprived)	6026 (25.5%)	4923 (26.1%)	1103 (23.1%)
2	6087 (25.7%)	4923 (26.1%)	1164 (24.3%)
3	4873 (20.6%)	3875 (20.5%)	998 (20.9%)
4	3941 (16.7%)	3072 (16.3%)	869 (18.2%)
5 (most deprived)	2733 (11.6%)	2084 (11.0%)	649 (13.6%)
Missing (*n*)	9	8	1
Comorbidities			
Chronic pulmonary disease	2716 (11.5%)	2033 (10.8%)	683 (14.3%)
Diabetes	1456 (6.2%)	1141 (6.0%)	315 (6.6%)
Renal disease	1076 (4.5%)	914 (4.8%)	162 (3.4%)
Cerebrovascular disease	650 (2.7%)	498 (2.6%)	152 (3.2%)
Congestive heart disease	345 (1.5%)	287 (1.5%)	58 (1.2%)
Peripheral vascular disease	264 (1.1%)	211 (1.1%)	53 (1.1%)
Myocardial infarction	250 (1.1%)	199 (1.1%)	51 (1.1%)
Peptic ulcer disease	238 (1.0%)	179 (0.9%)	59 (1.2%)
Liver disease	45 (0.2%)	35 (0.2%)	10 (0.2%)
Hot flushes	1693 (7.2%)	1024 (5.4%)	669 (14.0%)
Pre-diagnosis confounder medication use			
Hormone replacement therapy	7002 (29.6%)	5233 (27.7%)	1769 (37.0%)
Oral contraceptive	6009 (25.4%)	4454 (23.6%)	1555 (32.5%)
Treatment			
Surgery	19,218 (81.2%)	15,212 (80.6%)	4006 (83.7%)
Tamoxifen	10,051 (42.5%)	8006 (42.4%)	2045 (42.7%)
Radiotherapy	8320 (35.2%)	6683 (35.4%)	1637 (34.2%)
Chemotherapy	6774 (28.6%)	5213 (27.6%)	1561 (32.6%)
Aromatase inhibitors	4975 (21.0%)	4094 (21.7%)	881 (18.4%)
Grade			
1	3780 (17.9%)	2988 (17.8%)	792 (18.5%)
2	10,282 (48.7%)	8210 (48.8%)	2072 (48.3%)
3	7039 (33.3%)	5616 (33.4%)	1423 (33.2%)
4	19 (0.1%)	18 (0.1%)	1 (0.0%)
Missing (*n*)	2549	2053	496
Stage			
1	4791 (49.0%)	3812 (48.8%)	979 (49.7%)
2	3936 (40.2%)	3127 (40.0%)	809 (41.1%)
3	748 (7.6%)	623 (8.0%)	125 (6.4%)
4	309 (3.2%)	254 (3.2%)	55 (2.8%)
Missing (*n*)	13,885	11,069	2816
Smoking			
No	12,975 (61.3%)	10,653 (63.4%)	2328 (53.2%)
Ex	4677 (22.1%)	3696 (22.0%)	976 (22.3%)
Yes	3526 (16.6%)	2457 (14.6%)	1068 (24.4%)
Missing (*n*)	2491	2079	412
Alcohol			
No	3406 (19.7%)	2711 (19.7%)	697 (19.5%)
Ex	347 (2.0%)	252 (1.8%)	95 (2.7%)
Yes	13,577 (78.3%)	10,788 (78.5%)	2787 (77.9%)
Missing (*n*)	6339	5134	1205
Obesity			
Normal	7723 (41.0%)	6178 (41.5%)	1548 (39.3%)
Overweight	6326 (33.6%)	4999 (33.6%)	1325 (33.6%)
Obese	4776 (25.4%)	3707 (24.9%)	1068 (27.1%)
Missing (*n*)	4844	4001	843

Data presented as *n* (%) unless otherwise indicated

*SSRI* selective serotonin reuptake inhibitor, *SD* standard deviation

### Association between antidepressant use and survival

SSRI users were at a higher risk of breast cancer death than SSRI non-users (adjusted HR = 1.27; 95% CI 1.16, 1.40; Table 2) after adjustment for demographics, comorbidities and pre-diagnosis use of hormone replacement therapy or oral contraceptives. Although there was no evidence of a strong dose–response relationship, as patients receiving between 1 and 1095 DDDs had similar mortality, those receiving more than 1095 DDDs were at substantially greater risk of death (adjusted HR = 1.54; 95% CI 1.15, 2.07) than non-users. We found higher HRs of between 1.24 and 1.28 for citalopram, fluoxetine and paroxetine compared to sertraline (adjusted HR = 0.95; 95% CI 0.73, 1.23; Additional file 1: Appendix 3).

**Table 2:** Association between antidepressant use and breast cancer mortality

	All patients (*N* = 23,669)					Prior depression (*N* = 5417)^a^				
	*n*	Person-years	Deaths	Unadjusted HR (95% CI)	Adjusted HR^b^ (95% CI)	*n*	Person-years	Deaths	Unadjusted HR (95% CI)	Adjusted HR (95% CI)
All SSRIs combined										
Never	18,885	105,433	2539	Reference	Reference	2861	15,659	435	Reference	Reference
Ever	4784	20,722	514	1.17 (1.07, 1.29)	1.27 (1.16, 1.40)	2556	11,216	294	1.09 (0.93, 1.26)	1.14 (0.98, 1.33)
1–364 DDDs	2502	12,100	321	1.19 (1.06, 1.34)	1.28 (1.14, 1.44)	1055	5357	160	1.10 (0.92, 1.32)	1.15 (0.96, 1.38)
365–1094 DDDs	1176	4992	109	1.02 (0.84, 1.24)	1.12 (0.93, 1.36)	701	3167	68	0.87 (0.67, 1.13)	0.91 (0.70, 1.19)
1095–1824 DDDs	487	1847	47	1.37 (1.03, 1.84)	1.54 (1.15, 2.07)	333	1285	37	1.46 (1.04, 2.07)	1.58 (1.12, 2.24)
1825+ DDDs	619	1782	37	1.32 (0.95, 1.84)	1.53 (1.10, 2.13)	467	1407	29	1.37 (0.92, 2.03)	1.54 (1.03, 2.29)
Tricyclic antidepressants										
Never	18,734	104,636	2505	Reference	Reference	3140	16,608	463	Reference	Reference
Ever	4935	21,519	548	1.25 (1.14, 1.38)	1.30 (1.18, 1.43)	2277	10,267	266	1.08 (0.92, 1.25)	1.07 (0.92, 1.25)
1–364 DDDs	4041	17,989	464	1.24 (1.12, 1.37)	1.29 (1.17, 1.43)	1583	7509	203	1.05 (0.89, 1.24)	1.06 (0.89, 1.25)
365–1094 DDDs	574	2416	70	1.50 (1.18, 1.91)	1.51 (1.19, 1.92)	431	1865	53	1.32 (0.99, 1.77)	1.27 (0.94, 1.70)
1095–1824 DDDs	170	661	9	0.82 (0.43, 1.58)	0.94 (0.49, 1.80)	140	521	6	0.65 (0.29, 1.46)	0.68 (0.30, 1.52)
1825+ DDDs	150	454	5	0.75 (0.31, 1.81)	0.87 (0.36, 2.10)	123	372	4	0.71 (0.26, 1.92)	0.77 (0.29, 2.08)
Venlafaxine										
Never	22,826	122,227	2968	Reference	Reference	4976	24,830	684	Reference	Reference
Ever	843	3928	85	1.02 (0.82, 1.26)	1.30 (1.04, 1.61)	441	2046	45	0.94 (0.69, 1.27)	1.03 (0.76, 1.40)
1–364 DDDs	522	2531	66	1.19 (0.93, 1.51)	1.64 (1.28, 2.10)	214	1068	29	1.08 (0.74, 1.56)	1.32 (0.91, 1.92)
365–1094 DDDs	161	861	11	0.60 (0.33, 1.09)	0.67 (0.37, 1.21)	105	557	9	0.67 (0.35, 1.29)	0.66 (0.34, 1.28)
1095–1824 DDDs	68	293	2	0.36 (0.09, 1.43)	0.36 (0.09, 1.45)	47	227	1	0.22 (0.03, 1.57)	0.20 (0.03, 1.42)
1825+ DDDs	92	243	6	1.55 (0.70, 3.46)	1.76 (0.79, 3.94)	75	194	6	1.94 (0.86, 4.37)	2.05 (0.91, 4.63)

*HR* hazard ratio, *CI* confidence interval, *SSRI* selective serotonin reuptake inhibitor, *DDD* defined daily dose

^a^Restricted to patients with a diagnosis of depression, or prescription of an antidepressant, in the year prior to cancer diagnosis

^b^Adjusted for age, deprivation, year of diagnosis, cancer treatment within 6 months (radiotherapy, chemotherapy, surgery, tamoxifen, aromatase inhibitors), comorbidities (cerebrovascular disease, chronic pulmonary disease, congestive heart disease, diabetes, liver disease, myocardial infarction, peptic ulcer disease, peripheral vascular disease, renal disease), hot flushes and pre-diagnosis use of hormone replacement therapy or oral contraceptives

We also found higher breast cancer mortality among users of tricyclic antidepressants (adjusted HR = 1.30; 95% CI 1.18, 1.43; Table 2) when compared to tricyclic antidepressant non-users and venlafaxine (adjusted HR = 1.30; 95% CI 1.04, 1.61) when compared to venlafaxine non-users, but there was no clear dose–response relationship. The association between antidepressant use and breast cancer-specific mortality was attenuated substantially when restricting to patients with a prior diagnosis of depression (adjusted HR = 1.14; 95% CI 0.98, 1.33), although mortality remained much higher among long-term (> 1095 DDD) SSRI users when compared to SSRI non-users. HRs were also attenuated for tricyclic antidepressants (adjusted HR = 1.07; 95% CI 0.92, 1.25) and venlafaxine (adjusted HR = 1.03; 95% CI 0.76, 1.40), with no apparent dose–response relationship.

### Sensitivity and subgroup analyses

Our results were similar in the simpler analyses basing SSRI use on the first year after diagnosis or the year prior to diagnosis (Table 3). They were robust to changes in the exposure lag period from 6 months to 2 years, when expanding our breast cancer-specific death definition to include secondary causes, when omitting ‘previous hot flush diagnosis’ from the case-mix adjustment, for all-cause mortality, and did not change appreciably when adjusting for tumour prognostic features (i.e. stage, grade) or patient lifestyle factors (i.e. smoking, alcohol, obesity) using complete case or multiple imputation methods. There were small differences in the association between SSRI and cancer-specific mortality in tamoxifen users (adjusted HR = 1.36; 95% CI 1.15, 1.61), adherent tamoxifen users (adjusted HR = 1.30; 95% CI 1.03, 1.65) or tamoxifen non-users (adjusted HR = 1.25; 95% CI 1.11, 1.41). The association between SSRI use and breast cancer mortality was attenuated substantially when comparing to patients receiving a different antidepressant medication (adjusted HR = 1.06; 95% CI 0.93, 1.20).

**Table 3:** Sensitivity and subgroup analysis for SSRI use and breast cancer mortality

	Non-users^a^			Users			Unadjusted HR (95% CI)	Adjusted HR^b^ (95% CI)
	*n*	Person-years	Deaths^c^	*n*	Person-years	Deaths		
Main analysis	18,885	105,433	2539	4784	20,722	514	1.17 (1.07, 1.29)	1.27 (1.16, 1.40)
Death definition								
All-cause	18,885	105,433	4358	4784	20,722	933	1.16 (1.08, 1.25)	1.33 (1.23, 1.43)
Primary or secondary breast cancer cause	18,885	105,433	2965	4784	20,722	593	1.16 (1.06, 1.27)	1.28 (1.17, 1.40)
Exposure definition								
Year before diagnosis	21,768	138,969	2810	1901	10,855	243	1.09 (0.96, 1.24)	1.18 (1.03, 1.34)
Year after diagnosis	20,999	113,219	2700	2670	12,935	353	1.11 (1.00, 1.25)	1.23 (1.10, 1.38)
Exposure lag								
6 months	18,541	114,762	2455	5128	23,195	598	1.25 (1.14, 1.36)	1.36 (1.24, 1.49)
2 years	19,635	87,457	1912	4034	16,319	360	1.14 (1.02, 1.28)	1.22 (1.09, 1.37)
Prior antidepressant medication^d^	1769	9316	258	2019	8861	241	1.11 (0.93, 1.33)	1.21 (1.00, 1.45)
SSRI vs other antidepressant medication	3791	17,390	435	4784	20,722	514	1.02 (0.89, 1.15)	1.06 (0.93, 1.20)
Tamoxifen use								
Non-user	11,110	52,864	1674	2790	10,678	341	1.16 (1.03, 1.30)	1.25 (1.11, 1.41)
User	7775	52,569	865	1994	10,044	173	1.13 (0.96, 1.34)	1.36 (1.15, 1.61)
Adherant user^e^	3612	26,159	489	882	4645	86	1.10 (0.87, 1.38)	1.30 (1.03, 1.65)
Adjustment								
Excluding ‘previous hot flush’ diagnosis^f^	18,885	105,433	2539	4784	20,722	514	1.17 (1.07, 1.29)	1.24 (1.12, 1.36)
CC lifestyle^g^	12,568	70,673	1561	3290	14,116	327	1.18 (1.05, 1.33)	1.24 (1.10, 1.40)
MI lifestyle	18,885	105,433	2539	4784	20,722	514	1.17 (1.07, 1.29)	1.26 (1.14, 1.38)
CC diagnosis^h^	7388	40,419	813	1863	7980	164	1.13 (0.95, 1.34)	1.14 (0.96, 1.35)
MI diagnosis	18,885	105,433	2539	4784	20,722	514	1.17 (1.07, 1.29)	1.27 (1.14, 1.40)

*HR* hazard ratio, *CI* confidence interval, *SSRI* selective serotonin reuptake inhibitor

^a^Except for ‘SSRI vs other anti-hypertensive medication’ where patients who received a different anti-hypertensive medication serve as the reference group

^b^Adjusted for age, deprivation, year of diagnosis, cancer treatment within 6 months (radiotherapy, chemotherapy, surgery, tamoxifen, aromatase inhibitors), comorbidities (cerebrovascular disease, chronic pulmonary disease, congestive heart disease, diabetes, liver disease, myocardial infarction, peptic ulcer disease, peripheral vascular disease, renal disease), hot flushes and pre-diagnosis use of hormone replacement therapy or oral contraceptives

^c^Deaths with a primary cause of breast cancer unless otherwise stated

^d^SSRI users vs non-users, restricted to patients with an antidepressant prescription in the year prior to cancer diagnosis

^e^Defined as having been prescribed at least 150 defined daily doses of tamoxifen in the first six months after cancer diagnosis

^f^Not adjusting for a previous hot flush diagnosis

^g^Complete case (CC), additionally adjusted for smoking, obesity and alcohol consumption

^h^Multiply imputed (MI), additionally adjusted for stage and grade

## Discussion

### Summary of main findings

In this large, population-based cohort of newly-diagnosed breast cancer patients, we found that use of any SSRI was associated with a 27% increase in the risk of breast cancer mortality, and use for 3 years of more was associated with a 54% increase in mortality, after adjustment for patient demographics, comorbidities and pre-diagnosis use of hormone replacement therapy or oral contraceptives. These findings seem likely to reflect confounding by indication because the association was similar to those observed for other antidepressant medications, and was largely attenuated in additional analysis restricting to patients with a prior history of depression. However, additional studies are needed to further investigate the increase in mortality observed among long-term SSRI users.

### Strengths and weaknesses

Our study is based on a high-quality [35] population-based cohort of 23,669 patients with registry-confirmed breast cancer, and is nearly six times larger than previous work [31]. Patients included in the study had a long follow-up period after diagnosis of up to 17 years, which should allow any clinically important effect of SSRIs on breast cancer progression to become apparent. Linkage to ONS death registration data allowed robust verification of death, and facilitated a breast cancer-specific analysis, which should be more sensitive to small changes in disease-specific mortality and less susceptible to confounding by indication than all-cause deaths [40, 54]. Although some misclassification of death cause is possible, studies have shown this is likely to have a limited impact on our estimates (as there is no obvious mechanism for differential misclassification) [55] and our results were similar when including deaths where breast cancer was a secondary cause. We used prescribing data collected as part of routine clinical care which accurately reflects GP prescribing practices and negates the risk of recall bias. These data also included detailed information on the type of SSRI, and the strength, quantity and timing of prescription, which allowed us to investigate dose–response relationships and conduct separate analysis for specific medications. SSRIs are not available over-the-counter in the UK, which negates exposure misclassification due to over-the-counter use; however, SSRI prescriptions originating from secondary care are not captured within the CPRD.

Our study had several potential weaknesses. It is observational and hence open to confounding. Although we have adjusted for several of the key determinants of breast cancer progression (e.g. age, comorbidities and treatment), some other risk factors, including ethnicity and nutrition, were not available within our dataset [56, 57]. Furthermore, tumour stage and grade were missing for a large proportion of our cohort. Reassuringly, our findings were little altered when using multiple imputation to adjust for these factors. We do not know whether patients adhered to their SSRI medications; however, our main conclusions were similar when restricting our analysis to patients who received multiple prescriptions (e.g. > 1825 DDDs), where non-compliance is less of a concern. Lastly, we had limited data on hormone receptor status, although adjusting for tamoxifen and aromatase inhibitor use as proxy measures will limit the effect of this potential weakness.

### Comparison with previous research

We are aware of three previous studies, all based in the USA, which explored the impact of SSRI use on breast cancer patients regardless of their use of tamoxifen. The first study found that SSRI users had similar breast cancer mortality (adjusted HR = 1.0; 95% CI 0.4, 2.5) to non-users; however, as it was limited to only 1306 patients and 46 breast cancer deaths, it did not have sufficient statistical power to identify clinically meaningful differences between exposure groups [33]. The second study found higher breast cancer mortality among SSRI users (adjusted HR = 1.40; 95% CI 0.51, 2.79), which was consistent with our findings of a 27% increase. This study was also limited by its small sample size (*n* = 3058), reliance on self-reported questionnaires for medication use, poor response rate (36.2%) and inability to test dose–response relationships [32].

The third and most recent study, which used electronic prescribing records to determine SSRI use, death registries to identify cancer-specific deaths and robust case-mix adjustment for important prognostic factors (including stage), reported higher risk of breast cancer mortality among SSRI users (adjusted HR = 1.14; 95% CI 0.88, 1.48), consistent with our results. However, this study did not investigate dose–response relationships, had limited generalisability (due to its restriction to a single health insurance plan) and included substantially fewer patients than our study (*n* = 4216) [31]. Our analysis improves on previous work by using a large population-based dataset, with robust outcome and exposure assessment, to investigate breast cancer mortality by level of SSRI use. Our finding of higher mortality among long-term SSRI users, even after restricting to those with a prior diagnosis depression, is particularly novel and has not been explored previously.

Several studies have investigated SSRI use among tamoxifen users. One review concluded that SSRIs substantially inhibited the metabolism of tamoxifen [50], although individual studies have reached inconsistent conclusions [29, 30, 58]. Our finding of higher HRs among tamoxifen users is broadly consistent with previous work. However, the persistence of a substantial, albeit attenuated, association among patients who did not receive tamoxifen suggests that other mechanisms could be responsible.

### Implications for clinicians and researchers

The cause of higher cancer mortality among SSRI users remains unclear. We cannot rule out a causal relationship as preclinical studies have demonstrated that SSRIs increase prolactin levels [17–19], an accepted risk factor for breast cancer progression [20–22], and facilitate metastasis [23]. Although others have suggested that SSRIs could lead to poorer cancer outcomes through inhibition of the CYP2D6 enzyme among tamoxifen users [29], this was only partially supported by our study.

However, our study findings must be interpreted with caution as they are particularly vulnerable to confounding by indication, meaning that SSRI users had a higher underlying risk of mortality than the non-users. Depression, the main indication for SSRIs [14], has been shown to increase mortality in a meta-analysis of 18 studies of breast cancer patients [59], while a randomised controlled trial found reduced breast cancer mortality among patients assigned to a psychological intervention [60]. Although the mechanism for this association remains unclear, others have hypothesised that pathophysiological effects, through neuroendocrine and immunological functions, and a reduction in treatment adherence could play an important role [61]. Given our findings of similar associations in medications which have a weaker effect on prolactin levels or tamoxifen metabolism, and the substantial attenuation of these associations when restricting our cohort to patients with more similar diagnoses, it seems likely that confounding by indication is at least partly driving our results.

Our novel finding of increased cancer-specific mortality among long-term SSRI users requires further exploration, particularly as this association was not attenuated when restricting to those with prior depression diagnosis. It remains unclear whether this reflects a true effect of chronic prolactin overexposure on breast cancer progression, or, perhaps more likely, if issues with confounding by indication are exacerbated among patients with long-term depression.

## Conclusions

In this large population-based cohort of patients with registry-confirmed breast cancer, we found increased breast cancer mortality among SSRI users. However, this association was non-specific, and was substantially attenuated in additional analysis restricted to patients with more similar diagnoses. Consequently, clinicians should not be unduly concerned when prescribing SSRIs to breast cancer patients, but further research is needed into the effects of long-term SSRI use.

## Additional file

Additional file 1: Appendix 1. presenting a list of generic and proprietary drug names used to identify SSRIs, **Appendix 2** showing an illustration of the study design for selected analyses and **Appendix 3** presenting the association between SSRI use and breast cancer mortality for specific medications (DOCX 65 kb)

## Abbreviations

BMI: Body mass index
CPRD: Clinical Practice Research Datalink
DDD: Defined daily dose
HR: Hazard ratio
HRT: Hormone replacement therapy
IMD: Index of Multiple Deprivation
MICE: Multiple imputation with chained equations
NCDR: National Cancer Data Repository
ONS: Office for National Statistics
SSRI: Selective serotonin reuptake inhibitor
