# Supplementary material for: Selective serotonin reuptake inhibitor use and breast cancer survival: a population-based cohort study
Source: Breast Cancer Res. 2018 Jan 19;20:4. doi: 10.1186/s13058-017-0928-0 (PMC5775583; doi:10.1186/s13058-017-0928-0)
Supplement: Additional file 1: Appendix 1. — presenting a list of generic and proprietary drug names used to identify SSRIs, Appendix 2 showing an illustration of the study design for selected analyses and Appendix 3 presenting the association between SSRI use and breast cancer mortality for specific medications (DOCX 65 kb) [file 13058_2017_928_MOESM1_ESM.docx]

# Additional file 1

**Appendix 1: List of generic and proprietary drug names used to identify SSRI use**

| **Substance** | **Medication** |
| --- | --- |
| Citalopram | Citalopram, Cipramil |
| Escitalopram | Cipralex |
| Fluoxetine | Fluoxetine, Olena, Oxactin, Prozac |
| Fluvoxamine | Fluvoxamine, Faverin |
| Paroxetine | Paroxetine, Seroxat |
| Sertraline | Sertraline, Lustral |

**Appendix 2: Illustration of study design for selected analyses^a^**

*1 year*

DX Start FUP First SSRI End FUP

Main analysis

Sensitivity analysis: No exposure lag.

DX First SSRI End FUP

Start FUP

Sensitivity analysis: 2 year exposure lag.

DX Start FUP First SSRI End FUP

Sensitivity analysis: Year before diagnosis

SSRI DX End FUP

Start FUP

*Exposure Period, 1 year*

DX End FUP

Start FUP

*Exposure Period*

*1 year*

*2 years*

*2 years*

DX SSRI Start FUP End FUP

*Exposure Period, 1 year*

DX Start FUP End FUP

*Exposure Period*

Sensitivity analysis: Year after diagnosis

*Exposure Period*

*Exposure Period*

*Exposure Period*

SSRI User

SSRI Non-user

Before FUP

**Legend:**

^a^ FUP: follow-up period; DX: breast cancer diagnosis

**Appendix 3: Association between SSRI use and breast cancer mortality for specific medications**

|  | **N** | **Person-Years** | **Deaths** | **Unadjusted HR** | **Adjusted HR^a^** |
| --- | --- | --- | --- | --- | --- |
| **Citalopram** |  |  |  |  |  |
| Never | 20,731 | 114,975 | 2,775 | Ref | Ref |
| Ever | 2,938 | 11,180 | 278 | 1.18 (1.04,1.34) | 1.28 (1.13,1.45) |
| 1-364 DDDs | 1,769 | 7,535 | 188 | 1.15 (0.99,1.33) | 1.21 (1.04,1.41) |
| 365-1094 DDDs | 701 | 2,379 | 62 | 1.26 (0.98,1.62) | 1.43 (1.11,1.85) |
| 1095-1824 DDDs | 240 | 740 | 16 | 1.18 (0.72,1.94) | 1.40 (0.86,2.30) |
| 1825+ DDDs | 228 | 526 | 12 | 1.42 (0.80,2.50) | 1.81 (1.02,3.20) |
| **Fluoxetine** |  |  |  |  |  |
| Never | 22,144 | 118,761 | 2,879 | Ref | Ref |
| Ever | 1,525 | 7,394 | 174 | 1.13 (0.97,1.32) | 1.24 (1.06,1.45) |
| 1-364 DDDs | 913 | 4,750 | 115 | 1.13 (0.94,1.36) | 1.25 (1.04,1.51) |
| 365-1094 DDDs | 312 | 1,595 | 32 | 0.93 (0.66,1.32) | 1.01 (0.71,1.43) |
| 1095-1824 DDDs | 136 | 550 | 16 | 1.57 (0.96,2.57) | 1.73 (1.06,2.84) |
| 1825+ DDDs | 164 | 499 | 11 | 1.40 (0.77,2.53) | 1.56 (0.86,2.83) |
| **Sertraline** |  |  |  |  |  |
| Never | 22,785 | 123,150 | 2,995 | Ref | Ref |
| Ever | 884 | 3,004 | 58 | 0.91 (0.70,1.18) | 0.95 (0.73,1.23) |
| 1-364 DDDs | 526 | 1,838 | 41 | 1.03 (0.76,1.40) | 1.05 (0.77,1.43) |
| 365-1094 DDDs | 173 | 589 | 9 | 0.68 (0.35,1.30) | 0.71 (0.37,1.36) |
| 1095-1824 DDDs | 78 | 287 | 3 | 0.54 (0.17,1.66) | 0.63 (0.20,1.97) |
| 1825+ DDDs | 107 | 290 | 5 | 1.00 (0.41,2.40) | 1.11 (0.46,2.67) |
| **Paroxetine** |  |  |  |  |  |
| Never | 23,229 | 123,304 | 2,980 | Ref | Ref |
| Ever | 440 | 2,851 | 73 | 1.22 (0.97,1.54) | 1.26 (0.99,1.59) |
| 1-364 DDDs | 208 | 1,496 | 45 | 1.35 (1.00,1.81) | 1.38 (1.03,1.86) |
| 365-1094 DDDs | 106 | 750 | 12 | 0.75 (0.42,1.31) | 0.82 (0.46,1.44) |
| 1095-1824 DDDs | 41 | 269 | 8 | 1.56 (0.78,3.12) | 1.49 (0.74,2.98) |
| 1825+ DDDs | 85 | 337 | 8 | 1.51 (0.75,3.03) | 1.46 (0.73,2.94) |

^a^ Adjusted for age, deprivation, year of diagnosis, cancer treatment within 6 months (radiotherapy, chemotherapy, surgery, tamoxifen, aromatase inhibitors), comorbidities (cerebrovascular disease, chronic pulmonary disease, congestive heart disease, diabetes, liver disease, myocardial infarction, peptic ulcer disease, peripheral vascular disease, renal disease), hot flushes and pre-diagnosis use of hormone replacement therapy or oral contraceptives .
